# Supplementary material for: MetaQTL: a package of new computational methods for the meta-analysis of QTL mapping experiments
Source: BMC Bioinformatics. 2007 Feb 8;8:49. doi: 10.1186/1471-2105-8-49 (PMC1808479; doi:10.1186/1471-2105-8-49)
Supplement: Additional File 7 — MetaQTL Package : jar file and tutorial. This Zip archive contains both the MetaQTL JAR file and the files of the tutorial. [file 1471-2105-8-49-S7.zip › org.inra.metaqtl/doc/org/metaqtl/graph/MetaGraph.html]

MetaGraph


|  |  |  |
| --- | --- | --- |
| **Overview** \| **Package** \| **Class** \| **Use** \| **Tree** \| **Deprecated** \| **Index** \| **Help** | |  |
| **PREV CLASS**   **NEXT CLASS** | **FRAMES**    **NO FRAMES**     **All Classes** |  |
| SUMMARY: NESTED \| FIELD \| CONSTR \| METHOD | DETAIL: FIELD \| CONSTR \| METHOD |  |


---


## org.metaqtl.graph Class MetaGraph

```
java.lang.Object
  org.metaqtl.graph.MetaGraph
```

---

``` public class MetaGraph extends java.lang.Object ```

---

| **Field Summary** | |
| --- | --- |
| `org.metaqtl.graph.MetaGraph.CrossLayer[]` | `crossLayers` |
| `org.metaqtl.graph.MetaGraph.DataLayer[]` | `dataLayers` |
| `org.metaqtl.graph.MetaGraph.LegendLayer` | `legendLayer` |


**Constructor Summary**

`MetaGraph()`


| **Method Summary** | |
| --- | --- |
| `void` | `draw(java.awt.Graphics2D graph, double x, double y)` |
| `double` | `getHeight()` |
| `double` | `getWidth()` |
| `void` | `init(java.awt.Graphics2D graph, ChromGraph[] maps)` |

**Methods inherited from class java.lang.Object**

`clone, equals, finalize, getClass, hashCode, notify, notifyAll, toString, wait, wait, wait`

**Field Detail**

### dataLayers

```
public org.metaqtl.graph.MetaGraph.DataLayer[] dataLayers
```

---


### crossLayers

```
public org.metaqtl.graph.MetaGraph.CrossLayer[] crossLayers
```

---


### legendLayer

```
public org.metaqtl.graph.MetaGraph.LegendLayer legendLayer
```


**Constructor Detail**

### MetaGraph

```
public MetaGraph()
```


**Method Detail**

### draw

```
public void draw(java.awt.Graphics2D graph,
                 double x,
                 double y)
```

:   **Parameters:**: `graph` -: `x` -: `y` -: `sx` -: `sy` -

---


### init

```
public void init(java.awt.Graphics2D graph,
                 ChromGraph[] maps)
```

:   **Parameters:**: `graph` -: `maps` -

---


### getHeight

```
public double getHeight()
```

:   **Returns:**

---


### getWidth

```
public double getWidth()
```

:   **Returns:**


---


|  |  |  |
| --- | --- | --- |
| **Overview** \| **Package** \| **Class** \| **Use** \| **Tree** \| **Deprecated** \| **Index** \| **Help** | |  |
| **PREV CLASS**   **NEXT CLASS** | **FRAMES**    **NO FRAMES**     **All Classes** |  |
| SUMMARY: NESTED \| FIELD \| CONSTR \| METHOD | DETAIL: FIELD \| CONSTR \| METHOD |  |


---
